# Does Reproductive Success in Orchids Affect the Evolution of Their Number of Flowers?

**DOI:** 10.3390/plants14020204

**Published:** 2025-01-13

**Authors:** Iva Traxmandlová, Michaela Steffelová, Pavel Kindlmann

**Affiliations:** 1 Centre for Biology, Geoscience and Environmental Education, Faculty of Education, University of West Bohemia, Univerzitní 22, 30100 Pilsen, Czech Republic; 2 Institute for Environmental Studies, Faculty of Science, Charles University, Benátská 2, 12900 Prague, Czech Republic; misa.steffelova@atlas.cz (M.S.); pavel.kindlmann@centrum.cz (P.K.)

**Keywords:** fitness, model, orchid, reproductive success

## Abstract

Species are disappearing worldwide, and changes in climate and land use are commonly assumed to be the most important causes. Organisms are counteracting the negative effects of environmental factors on their survival by evolving various defence strategies, which positively affect their fitness. Here, the question addressed is: can evolution shape these defence strategies so that they positively affect the fitness of an organism? This question is complex and depends on the taxa and environmental factors. Therefore, here, only a special case of this question is studied in deceptive species of orchids: reproductive success (RS, ratio of the number of fruits to the number of flowers produced by a plant during the whole season), a commonly used measure of fitness is used to develop a model describing how RS affects the number of flowers, n, of a plant. This model predicts that: (i) the resulting relationship between RS and n is a positively skewed parabola, (ii) the distribution of the numbers of individuals with a specific number (n) of flowers, NI(n), also resembles a parabola and is also positively skewed, and that (iii) the peak of the distribution of NI is to the left of the peak of RS. A large set of data is presented that supports these predictions. If the data set is small, the concave positively skewed parabolic RS–n dependence is obscured by other factors.

## 1. Introduction

Species are disappearing worldwide, and it is predicted that this will become worse in the future [1]. The most important factors responsible for this are commonly assumed to be changes in climate and land use [2,3,4], with land use often ranked as the most important factor [5]. Land use change, mainly associated with the destruction of undisturbed or partially disturbed habitats, is the driving force of the biodiversity decline [6]. It is assumed that broad-scale global land-cover transformation will shortly increase species extinction rates [7,8,9,10]. Climate change is also often cited as one of the major driving forces [1,11,12,13,14,15,16,17]. There are, however, many other environmental factors that cause the extinction of species.

Organisms have evolved various strategies to counteract the negative effects of environmental factors on their reproductive fitness, which decrease the likelihood of extinction and might result in them becoming more abundant.

As this can be a very complex situation, depending on the taxa and environmental factors considered [18], this study focuses only on a small but very interesting aspect of this question: how reproductive success (RS, ratio of the number of fruits to the number of flowers produced by a plant during the whole season), a commonly used measure of fitness, affects the number of flowers, n, of a plant, and the probability distribution of the numbers of individuals with n flowers, NI(n), in deceptive orchids.

Orchids, our model group, belong to the family Orchidaceae, one of the most numerous families of flowering plants on Earth. In 2015, 736 genera were recorded [19]. The total number of orchid species is debatable; the estimated number of orchid species ranges between 24,000 and 28,000 species spread across approximately 880 genera [20,21,22]. They have very small seeds, with thousands, sometimes tens of thousands of seeds in one fruit (capsule)—[23,24], which is probably why the number of fruits is used rather than the number of seeds when plant fecundity is determined.

Orchids are divided into two groups according to the type of pollination. The flowers of the first group, which includes two thirds of the species, produce nectar as a reward for pollinators (“rewarding species”). Pollinators therefore visit them to receive the reward and thereby mediate the transfer of pollen. The second group consists of species with deceptive flowers (“deceptive species”) that deceive their pollinators by not providing nectar or any other reward, for example, pollen [25]. Deception appears to have evolved from a rewarding strategy that uses nectar [26] and occurs in roughly 9000 orchid species [22]. Other plants from other families also use deceptive flowers, yet orchids make up the majority of all deceptive plant species [27].

Deceptive species are characterized by special features that attract their pollinators [28,29]. Most often, these orchids have flowers that are visually similar in shape and colour to those of another species, which offers a reward in the form of nectar [27]. This is called Batesian mimicry [29]. In this case, the pollinator mistakes it for the flower of a rewarding species [30]. Such orchids include, for example, the species *Anacamptis pyramidalis*, *Anacamptis morio* and *Dactylorhiza majalis*, which are studied here. Other, less represented strategies are sexual mimics, scent, pollen, or imitations of anthers, shelter, or nectar [31].

RS is calculated as the ratio of the number of fruits to the number of flowers produced by a plant during one season [32,33]. It is a practical and easily determinable indicator of a plant’s potential success as RS affects the survival of a given species in subsequent years. For example, studies on *Habenaria* species revealed that fruit set and inflorescence size strongly influence reproductive output and, consequently, survival probabilities in fragmented habitats [34]. Species with a high RS are more likely to survive and less likely to become extinct. RS is affected by various factors, mainly the pollinators that visit flowers for a food reward: pollen, oil, or nectar [26]. Plants therefore invest in the production of flowers to attract pollinators because they ensure the transfer of pollen and thus allow the plant to reproduce, which is the only way a species can survive [35]. The main means by which pollinators are attracted is flower colour, fragrance, or the presence of a reward [36,37].

RS depends on many factors, which are often correlated and cannot be clearly distinguished: flower size, population size, population density, n, flowering phenology [38], height of plant or position of the flower within the inflorescence [39].

The RS of deceptive orchids can also be influenced by the time of flowering. Most orchids with deceptive flowers bloom earlier than those of rewarding orchids [40]. Sometimes, earlier flowering individuals have more fruits [41]. Species that bloom longer have more pollinated flowers than species that bloom for a shorter time [42,43]. To maximize RS and overall fitness, the plants adapt their flowering time in response to changing climatic conditions. RS can also be influenced by the vegetation zone and weather in the area where the orchid grows [44,45].

Rewarding species are more successful at attracting pollinators and have higher RS than deceptive species [46]. However, it is suggested that attractiveness may have evolved as a result of the higher fitness of the plant in a resource-scarce location [26]. By means of increasing its attractiveness, a plant does not have to invest as much energy in nectar production [26,47].

Some species of orchids benefit from having more flowers, even though they invest a lot of energy in them. More flowers produce a larger inflorescence that is more attractive to pollinators and the pollinator is more likely to spot the flower and see it from a greater distance [32,48,49]. Once a pollinator arrives at a deceptive orchid, it does not stay there for very long, because it does not receive a reward in the form of nectar [50,51]. In deceptive orchids, the pollinator visits only a few flowers (usually about three) in the inflorescence and then leaves the plant [50].

The RS of deceptive orchids is less than that of rewarding orchids [44]. Deceptive orchids can compensate for their low RS by having more seeds in the fruit, larger seeds, or more flowers. They conclude that deceptive orchids have on average more seeds per fruit than rewarding orchids [24]. Thus, deceptive orchids may survive despite having a low RS [27,31,52]. RS in deceptive orchids can be limited by a lack of resources, herbivory, or a shortage of pollinators [26].

The above raises important questions, e.g., what determines RS? How does n affect RS and if so, what is the shape of this dependence? These are the main questions addressed here.

Differences are reported in the dependence of RS on the n, both between genera and species. In some studies, no dependence of RS on the n is reported: in *D. majalis* and *D. maculata* [45], *Orchis ustulata* [40,45], *Phaius delavayi* [53], *Barkeria whartoniana* and *Cyrtopodium macrobulbon* [54]. In other species there is a positive dependence, as RS is larger for orchids with many flowers, for example in *Calopogon tuberosus* [55], *Myrmecophila christinae* [56], *D. incarnata* and *D. fuchsii* [40]. A concave parabolic dependence is reported for some species [40]. This may be because the data are very variable and the dependencies between the variables are not clear, and there is no or only an inconclusive mathematical model for this dependence [40,45,53,54,57].

Here, the aim is to explain when and why these dependencies occur, why they differ for different species and whether they result in changes in n.

## 2. Results

### 2.1. Model of the Dependence of RS on the n in Deceptive Orchids

Denote

- q—total number of plants in an area (site)
- n—number of flowers on a plant
- c—cost of production of one flower
- NI = NI(n)—number of individuals with n flowers, NI(n), in the population
- Poll—number of pollinators in the area
- p = p(n,k)—probability that k pollinators will visit a plant with n flowers
- m—min. No. of unsuccessful flower visits after which the pollinator leaves the plant
- k—parameter of the negative exponential
- RS(n,Poll)—reproductive success of a plant with n flowers, when Poll pollinators are present
- F—fitness of the plant (benefit minus cost)

**Figure 1:**
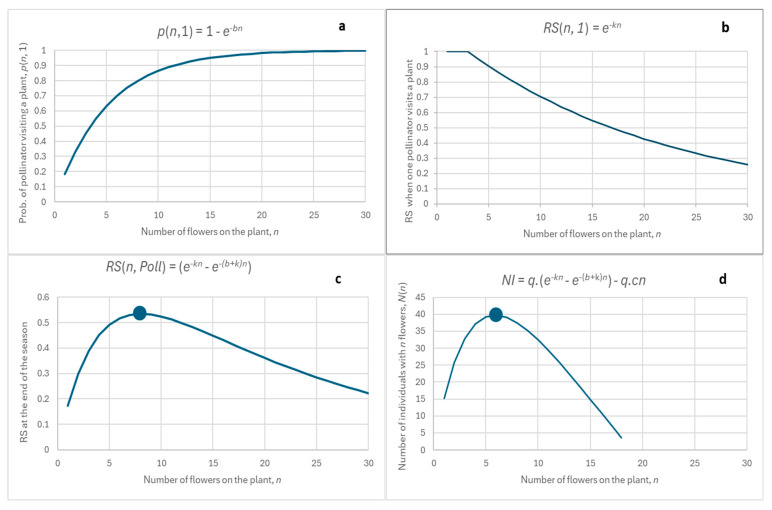
Results of the models (1)–(7): (**a**) the probability that a pollinator will visit a plant with n flowers during a season; (**b**) dependence of RS on the n, when one pollinator visits a plant during a season; (**c**) dependence of RS on then, when Poll pollinators visit a plant during a season; (**d**) number of plants with n flowers, NI(n), against the n of a plant, n, as predicted by (7). The blue dots mean the optimum number of flowers that maximizes RS—result of evolution (**c**) and number of individuals with this optimum number of flowers in the population (**d**), as predicted by the model. Equations used are at the top of each subfigure. Parameters used: b = 0.2, k = 0.05, q = 100, c = 0.02. Circles in (**c**,**d**) indicate peak of the curve.

Using the above notation, a mathematical function describing this situation was developed. In Figure 1, a hypothetical situation is depicted for a plant with a maximum of 30 flowers. The situation is as follows:

Probability and number of pollinator visits per plant: The probability that a pollinator will visit a plant with n flowers during a season is zero when n = 0 and this probability monotonously increases with increases in n, approaching the asymptote 1 for n approaching infinity. This probability is usually described as (Figure 1a):

p(n, 1) = 1 − e^−bn^ (1)

When Poll pollinators are in the area, then the expected number of pollinators that will visit a plant with n flowers during a season is therefore

Poll (n) = Poll (1 − e^−bn^) (2)

Reproductive success of a plant: When one pollinator visits a plant during a season then it is clearly: RS(n,1) = m/n (a hyperbola) because, for any n, exactly m flowers are pollinated.

When Poll pollinators visit a plant during a season, the situation becomes more complicated. RS is still the same, RS(n, Poll) = 1 for n < m, because by definition all flowers of a plant with m or fewer flowers are pollinated (see Figure 1b). However, for n > m, the total n pollinated on a plant with n flowers is not a simple product of m × k, but less, because different pollinators may visit the same flower. As it is unclear, what the overlap between pollinators pollinating the same flower might be, the best way to estimate RS is a commonly used curve for nonlinear decline—a negative exponential:

RS (n, Poll) = 1       for n < m(3a)

RS (n, Poll) = e^−kn^  for n > m (3b)

— see Figure 1b.

When the effect of plant attractivity for pollinators (Equation (2)) is also considered, then for n > m, the final RS_final_ is the product of (2) and (3):

RS_final_(n, Poll) = (e^−kn^ − e^−(b+k)n^) (4)

— see Figure 1c.

This RS is the benefit of the orchid strategy. The cost of this strategy is producing flowers:

Cost(n) = n·c(5)

The benefit minus cost value (the fitness, F, of the plant) is then the difference between (4) and (5):

F(n) = (e^−kn^ − e^−(b+k)n^) − cn (6)

The number of plants with n flowers in the population can be calculated from (6) by multiplying it by the total number of plants in the area, q:

NI (n) = q·(e^−kn^ − e^−(b+k)n^) – q·cn (7)

— see Figure 1d.

Figure 1 shows the typical behaviour of the model:

(a) the dependence of RS on n is a positively skewed parabola (Figure 1c),
(b) the distribution of the numbers of individuals with n flowers, NI(n), also resembles a parabola and is also positively skewed (Figure 1d),
(c) the peak of the distribution of NI (Figure 1d) is to the left of the peak of RS (Figure 1c).

**Figure 2:**
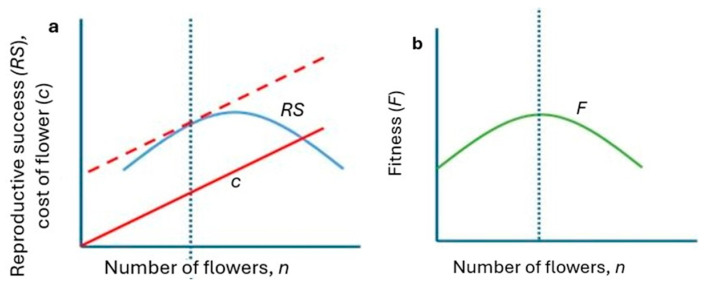
Graphical presentation of the situation: (**a**) dependence of RS on n for a plant, n (blue line), and dependence of the cost (energy required) of flower production on n (red line). The tangent parallel to the red line is the dashed red line. The point of contact of the dashed red line with the blue line determines the optimal n, which is the value of n, for which the difference between the benefit (blue line) and cost (red line) is maximal; (**b**) dependence of fitness, F, on n (benefit minus cost). benefit minus cost, green line.

Points (a)–(c) are not specific to this figure, which is presented here only for illustration. It can be easily mathematically proven that the shapes of the curves depicted in Figure 1 conform with (a)–(c) above. However, it exceeds the limits of this paper to prove it, and the reader is invited to check his/her mathematical skills and prove it independently. As a hint, Figure 2 shows a graphic presentation of the model’s predictions.

**Figure 3:**
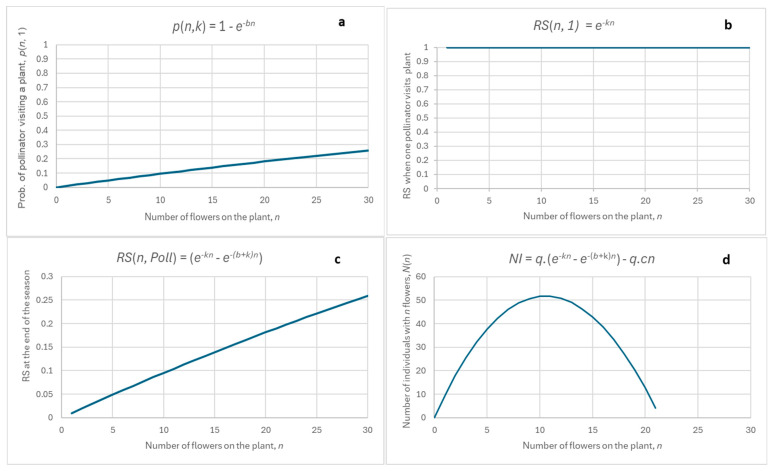
Results of the models (1)–(7): (**a**) the probability that a pollinator will visit a plant with n flowers during a season; (**b**) dependence of RS on n, n, of a plant when one pollinator visits it during a season; (**c**) dependence of RS on n, of a plant when Poll pollinators visit it during a season; (**d**) number of plants with n flowers, NI(n), against n of a plant, n, as predicted by (7). Equations used are at the top of each figure. Parameters used: b = 0.01, k = 0, q = 10,000, c = 0.009.

Interestingly, when certain special parameter values are used, the model predicts a very unexpected behaviour, as shown in Figure 3:

(1) the dependence of RS on n is positive and almost linear (Figure 3c), as opposed to the positively skewed parabola in Figure 1c.
(2) the distribution of the numbers of individuals with n flowers, NI(n), still resembles a parabola (Figure 3d).

This special case is mentioned because this type of behaviour occurs in *A. pyramidalis* and is reported below and a detailed explanatory model for this will be presented in the Discussion.

### 2.2. Fitting Empirical Data to the Model Predictions

**Figure 4:**
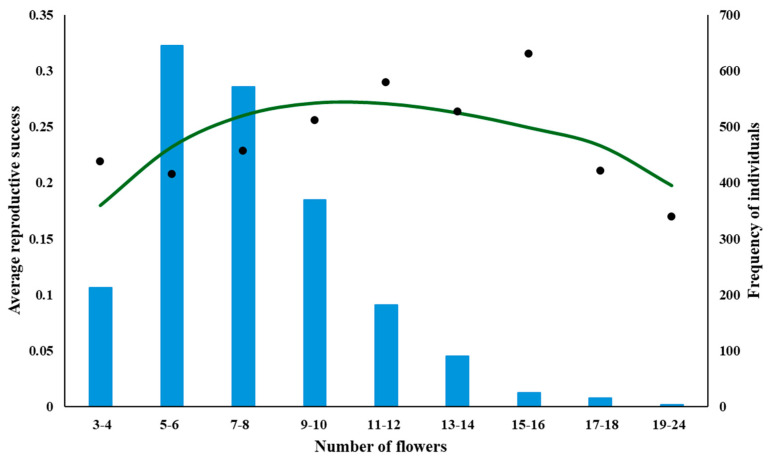
Dependence of RS on n for *A. morio*, fitted by the equation RS (n, Poll) = (e^−kn^ − e^−(b+k)n^)—see Table 1 for parameter values and dependence of the number of (NI) on n for these plants (blue bars). The plants were grouped based on the number of flowers (1–2, 3–4, 5–6, etc.).

**Figure 5:**
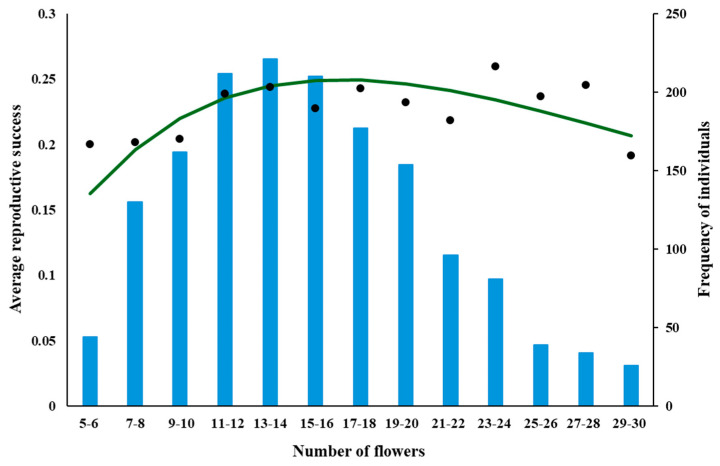
Dependence of RS on n for *D. majalis*, fitted by the equation RS (n, Poll) = (e^−kn^ − e^−(b+k)n^)—see Table 1 for parameter values and dependence of the number of individuals (NI) on n for these plants (blue bars). The plants were grouped based on the number of flowers (1–2, 3–4, 5–6, etc.).

**Figure 6:**
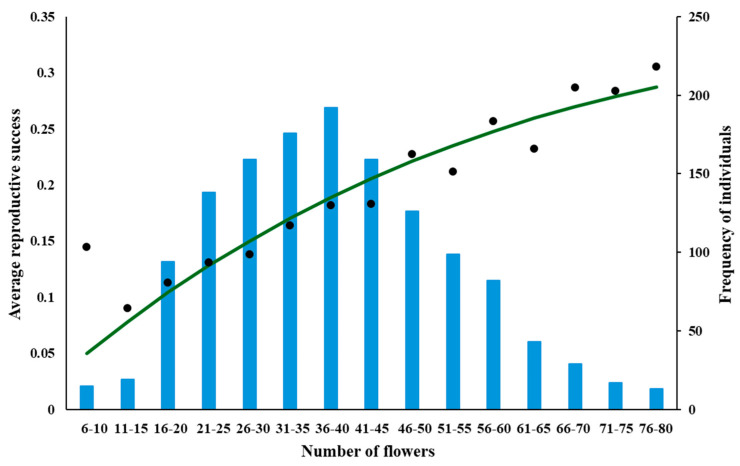
Dependence of RS on n for *A. pyramidalis*, fitted by the equation RS (n, Poll) = (e^−kn^ − e^−(b+k)n^)—see Table 1 for parameter values and dependence of the number of individuals (NI) on n for these plants (blue bars). The plants were grouped based on the number of flowers (1–5, 6–10, 11–15, etc.).

The data for *A. morio* and *D. majalis* indicates a concave, slightly positively skewed parabolic dependence of RS on n (Figure 4 and Figure 5), whereas for *A. pyramidalis* it is almost a linear dependence (Figure 6).

The dependencies of the frequencies of individuals on the number of their flowers are shown on the secondary axes of Figure 4, Figure 5 and Figure 6. The peaks of these dependencies were smaller than (to the left of) the peaks of the corresponding curves describing the dependence of RS on n for both *A. morio* and *D. majalis*, which is what the model predicts. The most frequently occurring individuals in terms of the number of flowers were those with 5–6 flowers in *A. morio*, 13–14 flowers in *D. majalis*, and 36–40 in *A. pyramidalis*.

## 3. Discussion

The reproductive success of orchids is closely linked to the availability of specific pollinators and environmental conditions, which are increasingly influenced by climate change. Studies show that climate change, particularly through temperature increases and changes in precipitation patterns, may disrupt these essential ecological interactions, posing a significant risk to orchid species. For example, in the case of the Australian orchid *Cryptostylis leptochila*, global warming is predicted to affect the availability of its specialized pollinator, the orchid dupe wasp (*Lissopimpla excelsa*), which is critical for its pollination success. Changes in climatic conditions, including droughts, can further exacerbate these disruptions, leading to reduced fruit production, a critical aspect of reproductive success [58].

Another study focusing on tropical orchids found that changes in flowering phenology due to shifting climatic conditions could lead to a mismatch between the timing of plant flowering and pollinator activity, reducing the effectiveness of pollination [59]. In addition, many orchid species are also narrowly adapted to specific habitats. Climate change is expected to shrink these suitable habitats, as seen in studies of orchids in Australia, where for instance, *Cryptostylis leptochila* is predicted to lose up to 76% of its habitat due to changes in temperature and precipitation patterns, despite potential new niches in cooler regions like Tasmania [58] or endangered orchids in the Qinghai–Tibetan Plateau [60]. The study [61] highlights the importance of habitat management and conservation efforts tailored to the ecological needs of specific orchid species. It also highlights the wider implications of habitat changes caused by anthropogenic activities or climate change for orchid populations.

**Figure 7:**
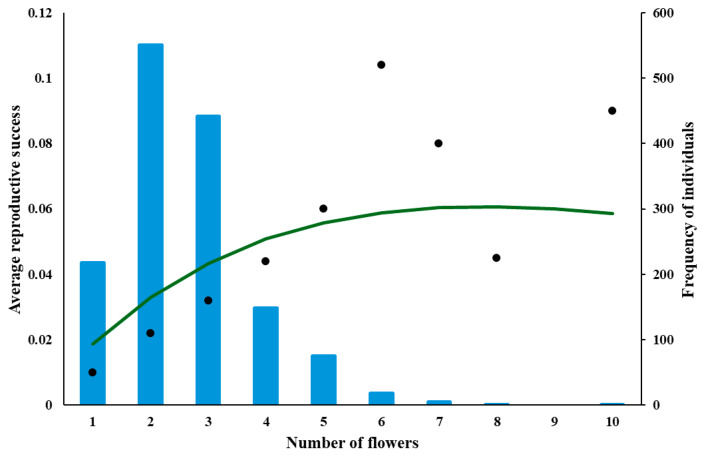
Dependence of RS on the n for *Calopogon tuberosus*, fitted using the equation RS (n, Poll) = (e^−kn^ − e^−(b+k)n^)—see Table 1 for parameter values, and the number of individuals (NI) with a given n (blue bars) (based on data in [54]).

*A. morio* and *D. majalis* in this study and *A. morio* in [40] have a concave positively skewed parabolic dependence of RS on n (RS–n dependence below). Biologically, this means it is not advantageous for these orchids to have too few or too many flowers: the most advantageous is to have a n somewhere between these two extremes. Consequently, evolution should result in the plants with the most advantageous n being most frequently represented in a population. As explained by the model, the cost of flower production must be considered, which results in the peak of the RS–n dependence being to the right of the peak of the NI–n dependence. This is supported by the results for *A. morio* and *D. majalis* in this study (Figure 3 and Figure 4) and those for *A. morio* and *D. majalis* in [40], in [62] for *A. morio* (see figure 8 in [62]), for *Calopogon tuberosus* ([55]—see the original and Figure 7 here) and for *D. majalis* in [63].

The RS–n dependence recorded for *A. pyramidalis* is surprisingly very different: almost linearly increasing, which is not exceptional in orchids as it is reported quite often in other studies (Table 2): in *D. incarnata* and *D. sambucina* [40] and in *Cephalanthera falcata* [64]. In other studies, it is claimed there is no dependence [39,53,64,65]. This large number of cases, which are contrary to the model’s predictions, which are supported by empirical results for two out of the three species studied is worrying and must be explained.

### 3.1. Does the RS–n Dependence Exist or Not? If Yes, What Is Its Shape?

Table 2 provides one possible explanation. In studies that indicate a concave positively skewed parabolical RS–n dependence (bold type in Table 2), i.e., *A. morio* and *D. majalis* in this study, *A. morio* in [39], and several other studies (see Table 2), the number of plants, in which RS was determined, was much higher (except [63]), several hundreds or thousands of individuals, compared with studies in which this dependence was positively linear or not observed (tens or low hundreds of individuals—*D. incarnata*, *D. fuchsii* and *O. ustulata* in [40,53,54,56,64,65]—see Table 2. However, there is a simple statistical explanation for this: large sample sizes generally result in greater precision when estimating unknown parameters. In other words, all things being equal, the smaller the effect, the greater the sample size needed to reveal it (see, e.g., The learning scientists, https://www.learningscientists.org/blog/2018/11/1-1 (accessed on 10 August 2024)). The low number of individuals measured may reveal a concave positively skewed parabolic RS–n dependence, but because it is weak, it is obscured by other factors.

The study of [55] based on 1457 individuals supposedly revealed a positive linear RS–n dependence (Figure 3 in their paper) as predicted by their model. However, their fit of a linear dependence is: (i) not good for large numbers of flowers (7, 8 and 10 flowers, see Figure 3 in their paper and Figure 7 presented here), and (ii) is strongly affected by a single value of 10 flowers for one individual. Statistically, therefore, their case is very weak. Repeating the regression (Figure 7) reveals a concave positively skewed parabola that is very similar in shape to a linear function (see Figure 7 here), which is in accord with the results presented here for two species and those of [40], in all these three datasets, thousands of individuals were measured. In addition, the peak of the NI-n dependence is to the left of the peak of the curve describing the RS–n dependence. All of these results are in accord with the model prediction presented in this paper.

### 3.2. Why Is the Shape of the RS–n Dependence Different in the Three Species Studied?

The data for *A. morio* and *D. majalis* reveal a concave positively skewed parabolic RS–n dependence (Figure 4 and Figure 5), which is consistent with the model’s predictions. That for *A. pyramidalis* reveals an almost linear RS–n dependence with a positive slope, which is significantly different from zero (*p* < 0.001) (Figure 6).

The model provides a possible explanation for the strange dependence recorded for *A. pyramidalis*: special parameter values were used in fitting the equation (see Figure 3), which were not rigorously verified against empirical data. In addition, there are several other biological explanations that should also be considered. These are:

(i) The three species studied differ in the duration and timing of flowering. *A. morio* and *D. majalis* bloom for two weeks, while *A. pyramidalis* blooms for up to four weeks and again two months later. Although precipitation and temperature may affect the duration of flowering and timing, the difference between *A. pyramidalis* and the other two species in this respect is significant and consistent [68]. It was also reported that the percentage pollination of an orchid, the flowers of which last a long time, is higher [42,43]. Also, late flowering may be associated with a larger number of pollinators and therefore a larger RS. This might result in the large RS for plants of *A. pyramidalis* with many flowers.
(ii) *A. morio* and *D. majalis* also have a significantly lower number of flowers and lower inflorescence density than *A. pyramidalis*. Orchids with more flowers and denser inflorescence are visited by more pollinators [42]. This may be an additional reason for the large RS of *A. pyramidalis* plants with the largest numbers (about 50–80) of flowers.
(iii) *A. pyramidalis* is significantly taller than *A. morio* and *D. majalis*. *A. pyramidalis* is pollinated mainly by butterflies [69] and *A. morio* and *D. majalis* mainly by bees [70,71]. All this may also contribute to the differences between these three species in the shape of the dependence of RS on n.

In summary: whether the strange behaviour of *A. pyramidalis* is due to special features of the model parameters (see Figure 3), or whether it should be attributed to other biological phenomena, like (i)–(iii), needs to be addressed in the future.

### 3.3. Other Aspects That May Influence the RS–n Dependence

It has been demonstrated that an increase in inflorescence size in *Brassavola nodosa* is followed by a stronger increase in RS than expected based on its n, which is not the case for *Calopogon tuberosus* [55,72]. All the flowers of *B. nodosa* open at about the same time, while those of *C. tuberosus* open sequentially, with typically only 2–4 flowers open at a time, keeping the effective inflorescence size small even if the inflorescences are large. This small effective inflorescence size in *C. tuberosus* may explain the lack of increase in RS above, expected for large inflorescences and indicates that the number of visits by pollinators per flower is probably more affected by the number of open flowers in an inflorescence than its size [55].

### 3.4. Summary and Ways Forward

None of the considerations in the last two subsections were studied. The explanations of why in many studies the concave parabolic dependence of RS on n was not revealed needs to be verified by further studies. An exception may be the studies presented in Table 2, in which there is no clear concave parabolic RS–n dependence, which is most likely due to the low number of plants included in these studies.

## 4. Materials and Methods

### 4.1. Species Studied

Orchids were used because they are very variable in many aspects: species richness [19,73,74], diversity of reproductive strategies [31], restricted distributions of small populations [18], and many are threatened by extinction [73,75,76]. Three species (*A. morio*, *A. pyramidalis* and *D. majalis*) were chosen as a representative sample of orchid populations because they occur in large numbers at certain sites, which is necessary for obtaining statistically significant measurements.

These three species with different life histories were chosen in order to generalize the results: *A. morio* grows on alkaline and acidic substrates with slightly moist to dry soils, *A. pyramidalis* prefers semi-steppe to wet meadows or open forests and calcareous substrates and *D. majalis* prefer wetland open habitats or semi-shaded places [77].

### 4.2. Sites Studied

*A. morio* was sampled at Svaté pole near Horažďovice, a relatively species-rich oat mesophilic meadow [78], which hosts tens of thousands of individuals of this species. *A. pyramidalis* was sampled in the protected landscape area (PLA) Český Kras, northeast of the city Srbsko, where *A. pyramidalis* grows on limestone in grassy meadows adjacent to a deciduous forest [79]. *D. majalis* was sampled in the first year on a mesophilic oat meadow near Brod northwest of Pilsen and, due to the scarcity of individuals there, also in a meadow near the village Číhaň southeast of Klatovy. In the following years, the data were collected only from the mesophilic oat meadow near Číhaň, which hosts several thousands of individuals of this species.

### 4.3. Data Collection

The data were collected every year for a period of three years (2021–2023). As the sites contained large populations of the species studied, always only one section of the site was selected at random, where all individuals were measured. The number of individuals measured is given in Table 3.

When the orchids were in bloom, each individual was marked with a red stick or stick with a red flag for easy finding of individuals after flowering. This prevented counting an individual more than once. For determining the RS of a plant, the n and capsules on each stem were counted after flowering.

RS was very variable due to variation in the numbers of pollinators: it often happens that for a given n, some plants are visited by many pollinators and others by very few or none [18,31]. Therefore, the plants were grouped based on the number of flowers (1–2, 3–4, 5–6, etc.) for *D. majalis* and for *A. morio*, and in groups of (1–5, 6–10, 11–15, etc.) for *A. pyramidalis* (because this species has many more flowers than the other two).

## Figures and Tables

**Table 1:** Parameters of the equation RS (n, Poll) = (e^−kn^ − e^−(b+k)n^) for each species.

Species	k	b	RSS	R^2^
*A. morio*	0.065	0.073	0.0094	0.42
*D. majalis*	0.042	0.042	0.0047	0.12
*A. pyramidalis*	0.0044	0.0067	0.012	0.81

**Table 2:** Overview of studies in which reproductive success was determined.

Species	Locality	No. of Ind.	R^2 1^	Shape ^2^	Region ^3^	References
*A. morio*	Svaté pole 2021–2023	2144	0.59	CA	TEMP	This study
*D. majalis*	Brod, Číhaň 2021–2023	1621	0.50	CA	TEMP	This study
*A. pyramidalis*	Srbsko	1393	0.92	PL	TEMP	This study
*D. majalis*	Páteříková Huť 2023	33	0.00	NT	TEMP	[66]
*D. fuchsii*	Páteříková Huť 2023	30	0.88	CV	TEMP	[66]
*D. majalis*	Cerhovice 2023	40	0.82	CV	TEMP	[67]
*D. majalis*	Pístovská louka 2022–2023	92	0.64	CA	TEMP	[63]
*A. morio*	Austria 2018	399	0.01	CA	TEMP	[62]
*Barkeria whartoniana*	2013	45	NC	NT	TROP	[54]
*Clowesia dodsoniana*	2013–2014	18	NC	NT	TROP	[54]
*Cyrtopodium macrobulbon*	2013–2014	43	NC	NT	TROP	[54]
*A. morio*	Zábrdí 1997	551	0.33	CA	TEMP	[40]
*A. morio*	Sirjansland 2000	202	0.00	NT	TEMP	[40]
*D. fuchsii*	Ohrazení 1994	52	0.39	CV	TEMP	[40]
*D. fuchsii*	Sv. Tomáš 1998	106	0.30	CV	TEMP	[40]
*D. fuchsii*	Gerendal 2000	121	0.33	CV	TEMP	[40]
*D. incarnata*	Kyselov 2000	152	0.40	PL	TEMP	[40]
*D. majalis*	Javorník 1998	100	0.00	NT	TEMP	[40]
*D. majalis*	Javorník 2000	164	0.00	NT	TEMP	[40]
*D. sambucina*	Javorník 2000	246	0.54	CA	TEMP	[40]
*D. sambucina*	Řetenice 2000	399	0.36	PL	TEMP	[40]
*O. ustulata*	Albrechtice 2001	84	0.00	NT	TEMP	[40]
*O. ustulata*	Vědlice 2001	137	0.00	NT	TEMP	[40]
*Calopogon tuberosus*	1980–1986	1457	0.50	CA	TEMP	[55]

^1^ R^2^: NC—not calculated; ^2^ Shape: CA—concave positively skewed parabolic dependence (corresponding studies in bold), CV—convex parabola, PL—positive linear, NT—no trend; ^3^ Region: TEMP—temperate, TROP—tropical.

**Table 3:** The number of individuals measured in a specific year.

Species		Year	
	2021	2022	2023
*Anacamptis morio*	492	600	1052
*Anacamptis pyramidalis*	387	407	599
*Dactylorhiza majalis*	531	500	590

## Data Availability

Data are contained within the article.
